# Hunting dogs bark differently when they encounter different animal species

**DOI:** 10.1038/s41598-021-97002-2

**Published:** 2021-09-23

**Authors:** Richard Policht, Ondřej Matějka, Kateřina Benediktová, Jana Adámková, Vlastimil Hart

**Affiliations:** Department of Game Management and Wildlife Biology, Faculty of Forestry and Wood Sciences, Czech University of Life Sciences Prague, Prague, Czech Republic

**Keywords:** Evolution, Zoology

## Abstract

Previous studies have shown that vocalizations of dogs can provide information to human companions. While acoustic signals of dogs have been intensively studied during the last decade, barking during hunting activities remains unstudied. The experiences of hunters indicate that owners can recognize what animal species has been found based on the bark of their dog. Such a phenomenon has never been tested before. We tested such an ability by comparing barks that were produced when dogs encountered four different animal species: wild boar, red fox, rabbit and fowl. Classification results of a discrimination analysis showed, that based on barks of dachshunds and terriers, it is possible to categorize towards which animal species barks were produced. The most distinctive barks were produced during encounters with the most dangerous of these animals, the wild boar. On the contrary, barks evoked by red fox encounters were classified similarly as those towards other smaller and non-dangerous animals like rabbits and fowl. Although the red fox represents a potentially dangerous species, the barking provoked was not classified with a much higher result than barking at animals that pose no threat. This might indicate that the key parameter could be the body size of the animal the dog meets. We further tested whether the degree of threat from the species of animal the dog encounters is reflected in the structure of the acoustic parameters based on the valence-arousal model. We found that barks produced in contact with a wild boar showed significantly lower frequency parameters and longest duration compared to other barks. According to these results, it seems that the variability of barking depending on the species of animal a dog encounters is an expression of the dogʼs inner state rather than functionally reference information.

## Introduction

Relationships between people and dogs, which represent the earliest domesticated animals, attract the attention of researchers in many ways^1–3^. The genetic history of dogs extends into the Palaeolithic, when at least five major ancestral lineages had diversified^4^. The exact timing of the emergence of the dog lineage remains unknown^5^. Current genetic studies estimate a time of dog–wolf divergence between 25,000 and 40,000 years ago^4,6^. Domestication of dogs caused differences from wolves in several ways. Adaptations included alterations in sequences of ritualized behaviour, and changes in motivational context for certain behaviours including changes in response thresholds^7,8^. Dogs are better at cooperating with humans than wolves are. They are more able to recognize our facial expressions and our communication signals; therefore, they work better with humans than wolves do^9^. Dog puppies display more communicative signals to facilitate social interactions, in contrast to wolf pups^9,10^. Thus, dogs show a unique complex of skills acquired for communication with humans^11^. Dogs bark frequently and use them in a wider range of contexts than their close relatives the wolf^12,13^ and coyote^12^, and the barks seem to have evolved from the low-frequency barks of wolves, which are mainly produced during agonistic interactions^14^. Barks changed quantitatively and qualitatively during the domestication process^15^. The complexity of the dog’s vocal repertoire has been extended by using mixed sounds in barking context. Various barking forms are generated via a mix of transitions and gradations of harmonics, intermediates and noisy subunits^3^. Some authors suggest that the original function of barks is mobbing (alerting other pack members and calling them in to defend the territory together)^16^. From this, hunting barks may have also be derived, as their function is to alert humans and lead them to the prey that the dog has found. Hunting-dog breeds were originally bred to fulfill some kind of hunting work. Humans artificially selected some breeds to bark frequently^14,17,18^. These facts indicate a strong selection force on barking performance in hunting dogs, which are also mentioned for several dog breed standards^14^. Additionally, sport-hunting breeds have been adapted to specific hunting work via an improved physiology, e.g. cardiac function, blood flow, and cognitive performance^19^. Some breeds have been bred for specific kinds of hunt, e.g. pointing breeds (pointers) were bred from dogs that were able to stand quietly and maintain its position in the face of the animal's scent until the human counterpart reaches the place where the animal is hiding. In contrast, other breeds were developed for multiple purposes or to be versatile and able to perform a number of tasks (e.g. hounds, retrievers, spaniels). Spaniels and retrievers will find and bring a shot animal to a hunter^20,21^. The dachshund is considered according to Fédération Cynologique Internationale one of the most versatile hunting breeds and not just for hunting below ground. Cooperative hunting dogs keep close contact with the hunter during the hunt (e.g. retrievers) whilst non-cooperative hunting dogs perform independent work, either chasing (e.g. beagles) or attacking (e.g. terriers) the animal^22^. Small terriers locate and hunt smaller mammals, while larger terriers are able hunt larger animals. Selected hunting breeds were bred to follow prey while barking, and some are even capable of specialised barking; on the other hand, other breeds have to stand silently and motionless near the found animal until the arrival of the hunter^20,23^. Specific forms of barking produced by some hunting dogs are even requested in the dog breed standards of international cynological organizations^14^. Recent studies have also shown that barks contain meaningful information based on context^17,24–27^, individual identity^24,26,27^, inner states^25^, and emotionality^28,29^.

We aimed to test whether hunting dogs produce barks differentially during encounters with different animal species. In order to test the barking of dogs at animals of different sizes, we needed to choose a universal dog breed. The choice of breeds for such a purpose was determined by the legislation of the Czech Republic. The Hunting Act distinguishes and defines four types of work performance. Dachshunds and terriers are the only groups of hunting dogs that can pass all four tests and be used for all types of hunting work in the Czech Republic. Although hounds are better suited to hunting wild boar, our law prohibits the use of dogs of a height of 55 cm or more for hunting ungulates. Dachshunds and terriers are no longer bred for earth-hunt work only, but are used for their independence and ability to adapt to surface work. For these reasons, dachshunds and terriers belong among the most common breeds for hunting all kinds of game in the Czech Republic. These breeds are considered to be independently working breeds that are able to work without visual contact with the hunter.

We used two different dog-breed groups: (A) dachshunds and (B) terriers. The hunting style of both breeds is as follows: looking for an animal, starting to bark, following in the footsteps of the animal, continuing to bark and chasing the animal to the hunter. In order to test potential bark differentiation, we recorded barks elicited by encounters with four different animal species. We selected animal models that would represent both (1) potentially dangerous animals (red fox, wild boar) and (2) non-dangerous animals (fowl, rabbit). Encounters with wild boars represent, for the small-bodied dog breeds used in our study, a real life-threatening situation accompanied by increased levels of emotion. Emotions with high-arousal are associated with a high sympathetic tone and a low parasympathetic tone^30^. Emotional arousal changes the muscular actions required for vocal production (e.g. diaphragm, vocal and intercostal muscles) which affect the way air flows through the vocal system and thus alter the quality of the sounds produced^31^. Expression of emotions informs other group members about the probable behavior intentions^32^. Vocal responses of dogs to these animal models could show us if acoustic structure of barks allow us to make predictions about how such signals change according to emotional arousal. Review on vocal correlates of emotions revealed that vocal signals of mammals become longer with increased arousal, louder and harsher, with higher and more variable frequencies and produced at faster rates^30^. Expression of emotions and perception of emotional states during hunting could play an important role in dogs as social species. Expression of emotions thus should benefit dogs by regulating social interactions within groups during hunting, whether it is a group of more dogs or dogs and hunters.

We have postulated the following hypotheses:

1. Barks produced during encounters with different animal species will have different acoustic structures. We can predict this based on previous robust literature showing that dog barks can be categorized based on various types of context^17,24^. Barks during hunting should vary with the demands of the situation, e.g. based on urgency or a common species-specific animal response, such as running away in hares, flight in pheasants, active defence in wild boar, etc.
2. Barks will show a different acoustic structure depending on the arousal of the caller. We predict that barks produced during the presence of non-dangerous species might differ from those produced in the presence of potentially dangerous species. We tested whether the degree of threat from the species of animal the dog encounters (e.g. wild boar versus rabbit) is reflected in the structure of the acoustic parameters based on the valence-arousal model^33^.

## Results

### Animal species context

**Figure 1:**
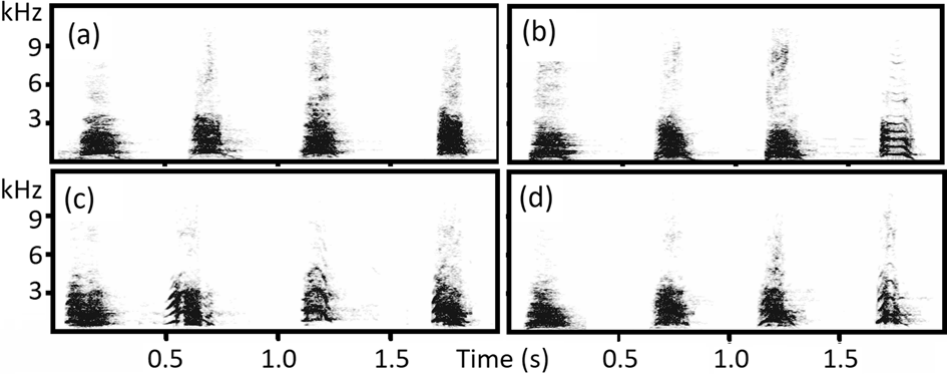
Spectrograms showing barking responses to wild boar, red fox, rabbit and fowl. Barks in each panel were produced by the same individual: Dachshunds Pecka (**a**) and Vendula (**b**). Fox Terriers Hard (**c**) and Gam (**d**).

We analyzed 1888 barks of 19 individual dogs belonging to two breeds—(1) dachsund and (2) terrier—which were produced in response to four different animal species (Fig. 1): wild boar, red fox, rabbit and fowl (Table 1). Figure 1 shows the bark spectrograms of the four types of barks (audio files: Additional Files 1–4).

**Table 1:** Tested dogs and number of analyzed barks per context.

Breed	Subject	Sex	Weight	Age	Wild boar	Red fox	Rabbit	Fowl
Dachshund	Hurvinek	M	7	6	30	30*	30*	30*
Dachshund	Amalka	F	5	4	0	30	30*	30*
Dachshund	Terezka	F	6	2	30	0	0*	0*
Dachshund	Nikolka	F	4	11	0*	30*	30*	30*
Dachshund	Venda-Benda	F	5	1	0	0	30*	30*
Dachshund	Vendula	F	7	10	30	30*	30*	30*
Dachshund	Kacka	F	5	4	30	30	30*	30*
Dachshund	Pecka	F	4	1	30	0	30*	30*
Dachshund	Punta	M	5	2	30	30	0*	30*
Fox Terrier	Gofi	F	7	2	0*	30*	30*	30*
Fox Terrier	Hard	M	10	1	30*	30*	30*	30*
Fox Terrier	Gam	M	9	2	30	30*	30*	30*
Fox Terrier	Cita	F	8	11	30*	30*	30*	0*
Fox Terrier	Bessy	F	8	7	30*	28*	30*	30*
Fox Terrier	Cerbis	M	10	3	30	30*	30*	30*
Fox Terrier	Nerys	M	9	9	0	30*	30*	30*
Fox Terrier	Ypsa	F	8	5	0*	30*	30*	30*
Welsh Terrier	Cit	M	7	3	30*	30*	30*	30*
Jagdterrier	Ar	M	10	2	30*	30*	30*	30*

*Previous experience with tested animal model. Dachshund Nikolka was castrated, everyone else was fertile.

To investigate whether acoustic parameters of barks differ in response to encountered animal species, we performed three separate stepwise discriminant function analyses (DFA) for three datasets (1) dachshunds only, (2) terriers only and (3) pooled data of both breeds.

The resulting models show a similar pattern for all three datasets independently (Table 2), including classification results (Table 3–4): the dachshund model (N = 9, n = 810, Wilks’ lambda = 0. 495, *p* < 0.001) included 8 acoustic parameters, the terrier model (N = 10, n = 1078, Wilks’ lambda = 0. 528, *p* < 0.001) included 10 parameters, and the pooled model (N = 19, n = 1888, Wilks’ lambda = 0. 549, *p* < 0.001) included 12 variables (Table 5). DFA for all three datasets showed that barks of dachshunds and terriers are recognizable based on the animal encountered with a higher probability than would be classified by chance (Table 2). The randomization procedure confirmed that these results were significant (pDFA, *p* < 0.001) for all three DFA models. Barks evoked by wild boar were classified better than those evoked by other animals (dachshund model: 60.6%; terrier model: 80.5%; pooled model: 73.3%), which is much higher than classification by chance (22.2%; 19.5% and 20.7% respectively) (Table 3). The percentages of correctly classified barks evoked by the other three animals were similar in all three models: dachshund model (43.3–52.1%), terrier model (27.8–56.2%), pooled model (35.7–49.8%). Classification outputs were significantly higher in comparison to classification by chance (dachshund model: Chi-Square = 128.7, df = 3, *p* < 0.001; terrier model: Chi-Square = 232.9, df = 3, *p* < 0.001; pooled model: Chi-Square = 170.9, df = 3, *p* <  0.001).

**Table 2:** The resulting discrimination function models. (Classif Orig/Valid /a priori) percentage of correct classification based on stepwise DFA, cross-validated DFA and a priori probability (classification by chance); (DF1, DF2) variable mostly correlated with the first and second discrimination function.

Result model	Classif Orig/Valid /a priori (%)			DF1 (correlation)	DF2 (correlation)
Dachshund	53.2	51.9	22.2–29.6	Duration (r = -0.49)	Q3T Rel (r = -0.43)
Terrier	52.8	51.1	19.5–27.9	Duration (r = 0.78)	Time 5% Rel (r = 0.57)
Pooled	50.9	49.6	20.7–27.0	Duration (r = -0.69)	Q1F Rel (r = 0.58)

**Table 3:** Confusion matrix for the animal species categorization task.

Result model	Rabbit	Fowl	Red fox	Wild boar	Prior probability
**Dachshund**					
Rabbit	**51.4**	32.9	2.9	12.9	25.9
Fowl	21.3	**52.1**	14.6	12.1	29.6
Red fox	10.6	33.9	**43.3**	12.2	22.2
Wild boar	13.9	23.9	1.7	**60.6**	22.2
**Terrier**					
Rabbit	**46.3**	19.3	27.3	7.0	27.9
Fowl	31.9	**27.8**	34.8	5.6	25.1
Red fox	23.6	14.8	**56.2**	5.4	27.6
Wild boar	10.5	1.4	7.6	**80.5**	19.5
**Pooled**					
Rabbit	**49.8**	24.9	16.3	9.0	27.0
Fowl	33.9	**35.7**	19.2	11.2	27.0
Red fox	21.0	23.9	**44.9**	10.3	25.3
Wild boar	9.0	11.0	6.7	**73.3**	20.7

Percentage of correct classification represents cross-validated results. A priori probability shows classification by chance (weighted by the number of analyzed barks). Bold numbers represent the percentage of correct classifications. Other values in rows show the percentages of incorrect classifications—that is, the percentage of barks wrongly classified as barks at another animal species.

**Table 4:** Classification results of bark subcategories in other studies. (Categ) classified categories, (No categ) number of classified categories, (Classif) correct classification percentage, (Chance) classification by chance, (Differ) difference between correct classification and classification by chance.

Categ	No categ	Breed	N Dogs	Method	Classif	Chance	Differ	Reference
Animal	4	Dachshund	9	DFA valid	51.9	25.0	28.2	This study
Animal	4	Terriers	10	DFA valid	51.1	25.0	26.9	This study
Context	7	Mudi	8	k-nearest	55.5	14.3	41.2	^59^
Context	6	Mudi		Humans listening	65–70	50	17.5	^35^
Context	6	Mudi	14	machine learning	43	18	25	^27^

**Table 5:** Measured acousticparameters.

Abbreviation	Name	Description	Units	DFA model
Duration	**Duration**	Signal duration	(s)	I,II,III
Time 5 Rel	**Time 5% relative**	The point in time that divides the signal into two time intervals containing 5% and 95% of the energy	(Rel)	I,II,III
Time 95 Rel	**Time 95% relative**	The point in time that divides the signal into two time intervals containing 95% and 5% of the energy	(Rel)	
Q1T Rel	**First quartile time relative**	The point in time that divides the signal into two time intervals containing 25% and 75% of the energy	(Rel)	II
Q3T Rel	**Third quartile time relative**	The point in time that divides the signal into two time intervals containing 75% and 25% of the energy	(s)	I,III
F5 Rel	**Frequency 5% relative**	The frequency that divides the signal into two frequency intervals containing 5% and 95% of the energy relative to frequency range	(Rel)	I,II
F5	**Frequency 5%**	The frequency that divides the signal into two frequency intervals containing 5% and 95% of the energy	(Hz)	II,III
F 95 Rel	**Frequency 95% relative**	The frequency that divides the signal into two frequency intervals containing 95% and 5% of the energy relative to signal duration	(Rel)	II
F 95	**Frequency 95%**	The frequency dividing the signal into two frequency intervals containing 95% and 5% of the energy	(Hz)	I
Q1F Rel	**First quartile frequency relative**	The frequency that divides the signal into two frequency intervals containing 25% and 75% of the energy relative to frequency range	(Rel)	I,II,III
Q1F	**First quartile frequency**	The frequency that divides the signal into two frequency intervals containing 25% and 75% of the energy	(Hz)	
Q3F	**Third quartile frequency**	The frequency that divides the signal into two frequency intervals containing 75% and 25% of the energy	(Hz)	III
CF	**Center frequency**	The frequency that divides the signal into two frequency intervals of equal energy	(Hz)	II, III
CT Rel	**Center time relative**	The point in time that divides a signal into two time intervals of equal energy	(Rel)	
IQRBW	**Inter-quartile Range**	The difference between the 1st and 3rd Quartile Frequencies	(Hz)	III
BW90	**Bandwidth 90%**	The difference between the 5% and 95% frequencies	(Hz)	III
Agg Entropy	**Aggregate Entropy**	The aggregate entropy measures the disorder (Bits) in a sound by analyzing the energy. Higher values correspond to greater disorder in the signal whereas a pure tone have zero entropy. It corresponds to the overall disorder in the sound.in a sound by analyzing the energy. Higher values correspond to greater disorder in the signal whereas a pure tone have zero entropy. It corresponds to the overall disorder in the sound	(Bits)	I,III
Avg Entropy	**Average Entropy**	The average entropy measures the average disorder in a sound. Describes the amount of disorder for a typical spectrum within the signal	(Bits)	II,III
Max Entropy	**Maximum Entropy**	This entropy is calculated by finding the entropy for each frame in the signal and then taking the maximum values	(Bits)	II,III
Min Entropy	**Minimum Entropy**	This entropy is calculated by finding the entropy for each frame and taking the minimum values	(Bits)	I

Measurements based on the Raven Pro manual. DFA model: (I) for dachshunds, (II) for terriers, and (III) pooled data of both breeds. The point in time that divides the signal into two specific time intervals is related to signal duration.

According to the arousal hypothesis it is expected that frequency parameters and call duration will differ between species which differ in their life-threatening level to hunting dog. We selected four frequency-related parameters (F5, Q1F, F95, Q3F) and call duration to test whether differences in the arousal state are encoded in barks.

**Figure 2:**
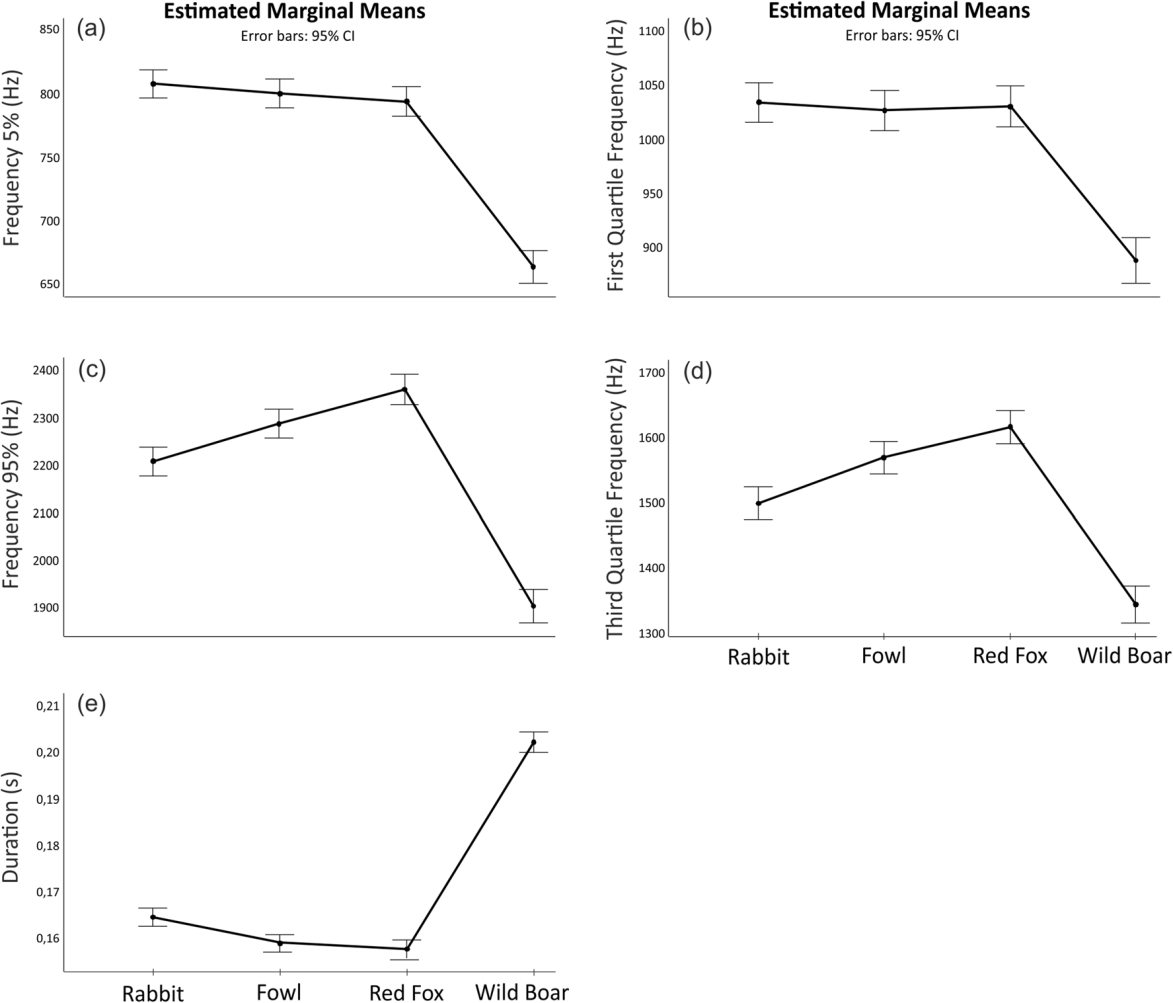
Univariate GLM comparison of acoustic parameters for arousal testing. Frequency parameters F5 and Q1F show the proportion of the acoustic energy in low frequencies (**a–b**), and F 95 and Q3F (**c**–**d**) show signal components in higher frequencies and temporal parameter Duration shows bark duration (**e**).

Barks in response to a wild boar showed significantly lower F5 than in response to other animals: F5 (GLM: F _3,18_ = 8.3, multiple comparisons: wild boar vs. all animals: *p* < 0.001). Differences between other animals (rabbit, fowl and red fox) were not significant (*p* ≥ 0.52), (Fig. 2). The lowest frequencies in the wild boar in comparison to other animals were also shown by the other three frequency parameters (Fig. 2).

Parameter F 95 showed significant differences between all animal pairs (GLM: F_3,18_ = 12.8, wild boar vs. all animals: *p* < 0.001, rabbit vs. red fox: *p* < 0.001, rabbit vs. fowl: *p*  = 0.002, red fox vs. fowl: *p* = 0.010).

Parameter Q3F also showed the biggest differences between wild boar and all other animals (GLM: F_3,18_ = 6.99, *p* < 0.001). The significant difference also showed comparison of the rabbit vs. fowl (*p* = 0.001) and rabbit vs. red fox (*p* < 0.001). Pair comparison of the red fox with rabbit and wild boar showed a significant difference (*p* < 0.001), while comparison with fowl was not significant (*p* = 0.073). Model of parameter Q1F did not show a significant effect of animal species (GLM: F_3,18_ = 1.52, *p*  = 0.222).

Temporal parameter Duration has been the longest in barks in response to a wild boar in comparison with all other animals (GLM: F _3,18_ = 32.4, *p* < 0.001). The comparison showed a significant difference for all other pairs (*p* < 0.001), only red fox and fowl did not differ (*p* = 1.0).

## Discussion

We aimed to test for potential differences in the barking of dogs when they encounter four different animal species—wild boar, red fox, rabbit and fowl—which represent models of various size and danger level for dogs. We used two groups of dogs—dachshunds and terriers. Classification results of a discrimination analysis showed that barks of dachshunds and terriers can be categorized based on the animal species they encountered with a higher probability than would be expected if classification was random. It was revealed that the most distinctive barks were made during encounters with the most dangerous animal, the wild boar. The same pattern was shown when we pooled both datasets together. On the contrary, barks evoked by red fox encounters were classified with a similar result to the other smaller and non-dangerous animals—here, the rabbit and fowl. Although the red fox represents a potentially dangerous species for small dog breeds, the provoked barks were not classified with a much higher success rate than barks at animals that pose no threat. This would indicate that the key parameter might be the body size of the animal the dog meets. When we compare the average success in the classification of barks at different animals with the classification results of different bark-classifying methods from different contexts we can see comparable results (see Table 4); however, different classification methods with different numbers of individuals and chance levels were used in these studies. When we take into account the resulting classification by chance level using weighting classification outputs by chance level, such a comparison may give us a general overview of the classification success generated by different classification methods.

The question is why hunting dogs bark differently at different animal species; is it because of a different inner state or it is a signal directed at their human companions? The barks investigated in previous studies were collected in different distinct social scenarios such as disturbance, isolation, play, presence of a stranger, fight training, begging, walk preparation, etc.^14,24^, and the acoustic structure of barks presumably reflects the inner states of dogs^34^ associated with these social contexts, which can be also recognized by humans^25,28,35^. Across these different contexts the emotional state of a dog may likely differ. We tested therefore whether the degree of threat from the species of animal the dog encounters (wild boar versus rabbit, red fox and fowl) can be reflected by the acoustic parameter structure according to the valence-arousal model^33^.

Barks produced during contact with a wild boar showed significantly lower frequency parameters. Frequency parameters seems to be not such a strong reliable indicator of emotions as they may both increase and decrease with an increase in arousal^30^, e.g. a shift in energy distribution towards higher frequencies was found in Weddel seal^36^, silver fox^37^, house cats^38^, red-fronted lemur^39^, squirrel monkey^40,41^, while towards lower frequencies in sheep^42^ and baboon^43^. Temporal parameters seem to be more consistent predictors of arousal. Vocalizations of mammals become longer with increase in arousal more frequently^30^. It is consistent with our result of the longest duration of barks produced during encounters with the most dangerous of tested animals, the wild boar.

In our case, it seems that the variability of barking, which depends on the species of animal the dog encounters, is an expression of a dog's inner state rather than functionally reference information. In addition, the expression of the inner state in barking appears to depend on the size of the potential threat. Barking in the case of a great threat (wild boar) is more specific than barking in the case of a smaller threat (red fox) or no threat (rabbit, fowl). This phenomenon could then indicate an innate ability, as it has been reported in the case of naive dogs, without previous experience with wild boar.

Both dog owners and non-owners, including adults and young children, are able to categorize a dog’s emotional state and barking context above the level of chance^17,24,25,28^. Domestic dogs bark frequently in comparison to feral dogs, which produce barks relatively rarely^44^. This fact could indicate that barking is at least in some way used for communication with humans^22^. Some authors have considered barks to be an exaggerated by-product of the domestication process that has no specific function^12^. Previous studies have shown that dog barks are able to express a wider range of emotions compared to those of wolves. Such a change in the acoustic communication of dogs has resulted from their association with humans^45^. Recognizing dog barks may be advantageous in inter-specific interactions since dog domestication occurred at least 30,000 years ago^46–48^. This process was initiated by European hunter-gatherers^47^. Mutualism between dog and hunter probably took place early after domestication^49^ when dogs assisted in the hunting of prey^47^. The ability to draw attention to different animal species complements hitherto known communicative skills like human–dog communication via eye contact^50^, changes in facial expressions of dogs affected by human attentional state^51^, the ability of dogs to understand the communicative cues of humans^52^ or communication using eye gaze^53,54^, and the widely known ability of dogs to understand human pointing gestures^52,55^.

Hunting dogs were bred to follow the trail of an animal. Some of these breeds were probably selected for a specific type of barking^14^. Such specialization could lead to the forming of an additional ability in comparison to other breeds not selected for hunting abilities. The hearing system of canids has primarily evolved to optimize predation, especially to localize sounds produced by potential prey^56^. Recognition of animal species could be favoured in reciprocal cooperation during hunting, e.g. recognition of potentially dangerous vs. non-dangerous animals could be especially favoured. Understanding the regulation mechanisms of mutual communication between humans and animals is especially important for animals such as dogs living in close contact with their human partners, depending on them for food, care and health^57^. The hunting activity of dogs with humans is considered to be derived from the cooperative behavior of wolves^22^. In hunting dogs, we might suppose that animal-encounter-specific barking may significantly increase the effectiveness of hunting events.

## Methods

### Ethics statement

This is a statement to confirm that all experimental protocols were approved by a named institutional or licensing committee. The authors declare that the present study complies with the current laws of the Czech Republic. The research was carried out in accordance with recommendations in the Guide for Care and Use of Animals of the Czech University of Life Sciences, Prague. This study focused on the recording of sounds, which was not considered an invasive experimental technique by The Professional Ethics Commission of the Czech University of Life Sciences Prague (project no 14/19) and did not require a special permit.

### Subjects

We recorded barks from 19 dogs (nine dachshunds—two males and seven females, eight fox terriers—four males and four females, one male Welsh terrier and one male jagdterrier) (Table 1) during December 2016 and March 2017. The age of both dachshunds and fox terriers ranged 1 to 11 years, the Welsh terrier was three years old and the jagdterrier two years old. Some dogs had previous experience with tested animal species and others were naive, with no previous experience (Table 1). The dog owners were coauthors of this study (KB and JA) and their colleagues.

### Experimental procedure

Recordings were conducted under semi-controlled conditions during outdoor experiments, not during huntig events. The experimental site was chosen in isolation from other objects and potential noise. No vegetation other than low grass was present during the winter and early spring. The experiments were performed in sunny weather without rainfall and almost no wind. Each dog was tested only once per day. The interval between experimental days was longer than fourteen days. Each of 19 dogs was randomly assigned to one of the four treatments (wild boar, red fox, rabbit and fowl). Recorded barks were elicited by encounters with live four different animal species through the fence mesh. There was no direct contact between the tested animals. Only one individual dog was tested during the experiment. Each tested dog was brought to the fence, released and left alone for 5–15 min depending on the frequency of barking required. The recording microphone was placed at a distance of two metres from the fence. We used an Olympus Linear PCM LS-5 audio-recorder with a Sennheiser ME 67 microphone (frequency response 20 Hz–20 kHz) with a K6 powering module.

### Acoustic analyses

We randomly selected a maximum of 30 barks per individual. These were chosen from a sample of barks of the best quality: non-overlapping barks with low background noise and a good signal-to-noise ratio. We did not obtain a full matrix as some dogs gave fewer than 30 barks. A total of 1888 barks were analyzed: 390 barks from the wild boar experiment, 508 barks from the red fox experiment, 510 barks from the rabbit experiment and 480 barks collected during the fowl experiment.We analyzed recordings using Raven Pro Sound Analysis Software (Cornell Lab of Ornithology, New York, USA) from which spectrograms were generated using the following parameters: Hann window type with a 1050 point window size, an overlap of 50%, a hop size of 11.9 ms, and grid spacing of 21 Hz. We measured 20 acoustical parameters (Table 5).

### Statistical analyses

From the measured parameters we excluded those that were highly correlated (r ≥ 0.90: F95 Rel and Avg Entropy), and the remaining variables were entered into the discriminant function analysis (DFA) (Table 3). We performed three types of analyses: (1) for dachshunds, (2) for terriers and (3) for both breeds together (pooled model). We performed a stepwise DFA in order to test whether dog barks can be classified based on the animal species that they were produced in response to. The procedure selected predictors using the Wilks’ lambda criterion. We used F values as a criterion for entering or removing an acoustic parameter from a classification model (F to enter = 3.84; F to remove = 2.71). For external validation of this model we used leave-one-out cross-validation using IBM SPSS 20 (IBM Corp., Armonk, USA). Animal species were used as a group identifier and the acoustic variables were used as discriminant variables. We normalized measured variables using Z score transformation (by subtracting the mean and dividing by the variable’s standard deviation), which avoids the false attribution of weights in relation to variables measured in different units (IBM Corp., Armonk, USA). We then performed a permuted DFA (pDFA) for nested designs, which serves as a randomization procedure for non-independent data^58^. We calculated pDFAs using a script written in software “R” (provided by Roger Mundry) using 100 random selections and 10,000 permutations. This procedure gave a p-value which was used to determine the significance of the correct classification rate of barks to the test factor (animal species), while controlling for a single nested factor (individual). N refers to number of individuals (dogs), n refers to number of calls (barks).We used univariate general linear models (GLM) for the motivational–structural test to see if barks differ between the animal species they were produced in response to. Acoustic variables were used as dependent variables, animal species as a fixed factor, and individual dogs as a random factor. We used Bonferroni corrected post hoc multiple comparison. It was also used Chi-Square test based on the observed versus expected values to test classification outputs of DFA models.

## Supplementary Information

Supplementary Information 1.

Supplementary Information 2.

Supplementary Information 3.

Supplementary Information 4.

Supplementary Information 5.

## Data Availability

The datasets generated during and analyzed during the current study are available from the corresponding author on request.

## References

[1] Yeomans L, Martin L, Richter T (2019). Close companions: early evidence for dogs in northeast Jordan and the potential impact of new hunting methods. J. Anthropol. Archaeol..

[2] Serpell J (2016). The Domestic Dog: Its Evolution, Behavior and Interactions with People.

[3] Jensen P (2007). Behavioural Biology of Dogs.

[4] Bergström A (2020). Origins and genetic legacy of prehistoric dogs. Science.

[5] Pavlidis P, Somel M (2020). Of dogs and men. Science.

[6] Botigué LR (2017). Ancient European dog genomes reveal continuity since the Early Neolithic. Nat. Commun..

[7] Bradshaw JWS, Lea AM (1992). Dyadic interactions between domestic dogs. Anthrozoös.

[8] Fox MW (1971). Behaviour of Wolves, Dogs and Related Canids.

[9] Gácsi M (2005). Species-specific differences and similarities in the behavior of hand-raised dog and wolf pups in social situations with humans. Dev. Psychobiol..

[10] Topál J (2005). Attachment to humans: a comparative study on hand-reared wolves and differently socialized dog puppies. Anim. Behav..

[11] Hare B, Brown M, Williamson C, Tomasello M (2002). The domestication of social cognition in dogs. Science (New York, N.Y.).

[12] Cohen JA, Fox MW (1976). Vocalizations in wild canids and possible effects of domestication. Behav. Proc..

[13] Siniscalchi M, Dingeo S, Minunno M, Quaranta A (2018). Communication in dogs. Animals (Basel).

[14] Pongrácz P, Molnár C, Miklósi Á (2010). Barking in family dogs: an ethological approach. Vet. J..

[15] Pongrácz P, Szabó É, Kis A, Péter A, Miklósi Á (2014). More than noise?—field investigations of intraspecific acoustic communication in dogs (Canis familiaris). Appl. Anim. Behav. Sci..

[16] Lord K, Feinstein M, Coppinger R (2009). Barking and mobbing. Behav. Proc..

[17] Yin S (2002). A new perspective on barking in dogs (*Canis familaris*). J. Comp. Psychol..

[18] Yeon SC (2007). The vocal communication of canines. J. Vet. Behav. Clin. Appl. Res..

[19] Kim J (2018). Genetic selection of athletic success in sport-hunting dogs. Proc. Natl. Acad. Sci..

[20] Lamb V (2006). The Ultimate Hunting Dog Reference Book: A Comprehensive Guide to More than 60 Sporting Breeds.

[21] Jakovcevic A, Elgier AM, Mustaca A, Bentosela M (2010). Breed differences in dogs' (*Canis familiaris*) gaze to the human face. Behav. Proc..

[22] Miklosi A (2007). Dog Behaviour, Evolution, and Cognition.

[23] Christiansen FO, Bakken M, Braastad BO (2001). Behavioural differences between three breed groups of hunting dogs confronted with domestic sheep. Appl. Anim. Behav. Sci..

[24] Yin S, McCowan B (2004). Barking in domestic dogs: context specificity and individual identification. Anim. Behav..

[25] Pongracz P, Molnar C, Doka A, Miklosi A (2011). Do children understand man's best friend? Classification of dog barks by pre-adolescents and adults. Appl. Anim. Behav. Sci..

[26] Molnár C, Pongrácz P, Faragó T, Dóka A, Miklósi Á (2009). Dogs discriminate between barks: the effect of context and identity of the caller. Behav. Proc..

[27] Molnar C (2008). Classification of dog barks: a machine learning approach. Anim. Cogn..

[28] Pongrácz P, Molnár C, Miklósi Á, Csányi V (2005). Human listeners are able to classify dog (*Canis familiaris*) barks recorded in different situations. J. Comput. Psychol..

[29] Pongrácz P, Molnár C, Miklósi Á (2006). Acoustic parameters of dog barks carry emotional information for humans. Appl. Anim. Behav. Sci..

[30] Briefer EF (2012). Vocal expression of emotions in mammals: mechanisms of production and evidence. J. Zool..

[31] Titze, I. R. Principles of voice production. Englewood Cliffs, NJ: Prentice Hall., (Prentice Hall, 1994).

[32] Panksepp, J. Emotional causes and consequences of social-affective vocalization. In *Handbook of mammalian vocalization – an integrative neuroscience approach* (ed Brudzynski, S. M.) 201–208 (Academic Press, London, 2009).

[33] Briefer, E. F. Coding for ‘Dynamic’ information: Vocal expression of emotional arousal and valence in non-human animals. In *Coding strategies in vertebrate acoustic communication* (eds Aubin, T. & Mathevon, N.) 137–162 (Springer International Publishing, Cham, 2020).

[34] Pongracz P (2017). Modeling evolutionary changes in information transfer: effects of domestication on the vocal communication of dogs (*Canis familiaris*). Eur. Psychol..

[35] Molnar C, Pongracz P, Doka A, Miklosi A (2006). Can humans discriminate between dogs on the base of the acoustic parameters of barks?. Behav. Proc..

[36] Collins K, McGreevy P, Wheatley K, Harcourt R (2011). The influence of behavioral context on Weddell seal (*Leptonychotes weddellii*) airborne mother-pup vocalization. Behav. Proc..

[37] Gogoleva S, Volodina E, Volodin I, Kharlamova A, Trut L (2010). The gradual vocal responses to human-provoked discomfort in farmed silver foxes. Acta Ethologica.

[38] Yeon SC (2011). Differences between vocalization evoked by social stimuli in feral cats and house cats. Behav. Proc..

[39] Fichtel C, Hammerschmidt K (2002). Responses of Redfronted lemurs to experimentally modified alarm calls: evidence for urgency-based changes in call structure. Ethology.

[40] Fichtel C, Hammerschmidt K (2003). Responses of squirrel monkeys to their experimentally modified mobbing calls. J. Acoust. Soc. Am..

[41] Fichtel C, Hammerschmidt K, Jürgens U (2001). On the vocal expression of emotion. A multi-parametric analysis of different states of aversion in the Squirrel monkey. Behaviour.

[42] Sèbe, F. *et al*. A. Bio-acoustic analyses to assess emotion in animals: acoustic patterns are linked to behavioural, cardiac and hormonal responses of ewes to the separation from their lambs. *Proceedings of the International Bioacoustics Council meeting, Lisbon*, Portugal. Biacoustics 51, 54. (2012).

[43] Meise K, Keller C, Cowlishaw G, Fischer J (2011). Sources of acoustic variation: implications for production specificity and call categorization in chacma baboon (*Papio ursinus*) grunts. J. Acoust. Soc. Am..

[44] Boitani L, Ciucci P (1995). Comparative social ecology of feral dogs and wolves. Ethol. Ecol. Evol..

[45] Miklosi, A. Human-animal interactions and social cognition in dogs. In *Behavioural biology of dogs *(ed Jensen, P.) 207-222 (CABI Publishing, Wallingford, UK, 2007).

[46] Grimm D (2016). Dogs may have been domesticated more than once. Science.

[47] Thalmann O (2013). Complete mitochondrial genomes of ancient canids suggest a European origin of domestic dogs. Science.

[48] Amici F, Waterman J, Kellermann CM, Karimullah K, Bräuer J (2019). The ability to recognize dog emotions depends on the cultural milieu in which we grow up. Sci. Rep..

[49] Clutton-Brock J (1987). A Natural History of Domesticated Animals.

[50] Kaminski J, Schulz L, Tomasello M (2012). How dogs know when communication is intended for them. Dev. Sci..

[51] Kaminski J, Hynds J, Morris P, Waller BM (2017). Human attention affects facial expressions in domestic dogs. Sci. Rep..

[52] Bräuer J, Kaminski J, Riedel J, Call J, Tomasello M (2006). Making inferences about the location of hidden food: Social dog, causal ape. J. Comput. Psychol..

[53] Soproni K, Miklosi A, Topál J, Csányi V (2001). Comprehension of human communicative signs in pet dogs (*Canis familiaris*). J. Comput. Psychol..

[54] Téglás E, Gergely A, Kupán K, Miklosi A, Topál J (2012). Dogs' gaze following Is tuned to human communicative signals. Curr. Biol..

[55] Miklosi A, Soproni K (2006). A comparative analysis of animals' understanding of the human pointing gesture. Anim. Cogn..

[56] Bradshaw, J. & Rooney, N. Dog social behavior and communication. In *The domestic dog: Its evolution, behavior and interactions with people. Second edition.* (ed Serpell, J.) (Cambridge University Press, 2016).

[57] Ntalampiras SA (2019). Automatic classification of cat vocalizations emitted in different contexts. Animals (Basel).

[58] Mundry R, Sommer C (2007). Discriminant function analysis with nonindependent data: consequences and an alternative. Anim. Behav..

[59] Larranaga A (2015). Comparing supervised learning methods for classifying sex, age, context and individual Mudi dogs from barking. Anim. Cogn..

