# Supplementary material for: Hunting dogs bark differently when they encounter different animal species
Source: Sci Rep. 2021 Sep 23;11:17407. doi: 10.1038/s41598-021-97002-2 (PMC8460642; doi:10.1038/s41598-021-97002-2)
Supplement: Supplementary file 5 — Supplementary Information 5. [file 41598_2021_97002_MOESM5_ESM.docx]

Supplementary Information

Hunting dogs bark differently when they encounter different animal species.

Richard Policht*, Ondřej Matějka, Kateřina Benediktová, Jana Adámková, Vlastimil Hart

Department of Game Management and Wildlife Biology, Faculty of Forestry and Wood Sciences, Czech University of Life Sciences Prague, Prague, Czech Republic

* Corresponding Author

**Supplementary Audio S1.** Four barks produced at wild boar, red fox, rabbit and fowl originating from the same individual (Dachshund Pecka).

**Supplementary Audio S2.** Four barks produced at wild boar, red fox, rabbit and fowl originating from the same individual (Dachshund Vendula).

**Supplementary Audio S3.** Four barks produced at wild boar, red fox, rabbit and fowl originating from the same individual (Fox Terrier Hard).

**Supplementary Audio S4.** Four barks produced at wild boar, red fox, rabbit and fowl originating from the same individual (Fox Terrier Gam).
